# The XXXVIII Long Fox Memorial Lecture: The Use of Tracer Elements in Biology and Medicine

**Published:** 1950-01

**Authors:** John E. Harris

**Affiliations:** Professor of Zoology in the University of Bristol


THE XXXVIII LONG FOX MEMORIAL LECTURE

*Delivered in the University of Bristol*
*on Tuesday, October 5th, 1949*

BY

JOHN E. HARRIS
*Professor of Zoology in the University of Bristol.*

ON

### THE USE OF TRACER ELEMENTS IN BIOLOGY AND MEDICINE

The term "tracer element" is used to describe elements which are chemically identical with, but physically different from the ordinary forms of elements occurring in nature. Their chemical identity implies that such elements take part in all the reactions of the living organism in exactly the same manner as does the normal counterpart; but their physical differences are a label by which we can follow their path through any system into which they may be introduced. Deuterium ("heavy" hydrogen) was one of the first of such elements to be extensively employed, and Schonheimer and Rittenberg in the U.S.A. brilliantly exploited its use from 1935 onwards in studies of metabolic changes. Organic compounds containing the heavy hydrogen isotope, when burnt, yield heavy water which can be estimated simply by determining its specific gravity.

Schonheimer and his colleagues were able to show that the metabolic changes occurring in the body were not a system of one-way traffic routes, in which nutrients (and water) went through a sequence of clearly defined chemical processes and finished up as some definite end product: but that most of the chemical products in the body tissues were taking part in a continuous ebb and flow of reactions, interchanging material unceasingly—a sort of physiological witches' cauldron, to which has been given the name of the "metabolic pool" of the organism. This pool takes in material—digested foodstuffs, water, oxygen, etc.—and releases waste products like carbon dioxide, and urea. But within it nothing is static; even the fats and proteins which are the building bricks out of which our bodies are made are continuously breaking down and being rebuilt.

In the study of such manifold and intricate changes tracer elements can play an increasingly important part. Certain elements (of which nitrogen is of fundamental interest) are only available for study in the form of stable isotopes. If only small amounts of the heavy isotope are present, or if its physical properties are very similar to those of the normal isotope, crude density measurements are inadequate to estimate its content. In such circumstances the mass spectrograph, a magnetic separator, is employed; this simultaneously sorts and estimates the relative amount of heavy isotope present in a sample.

By far the most spectacular of the isotopes used nowadays are those which are unstable, the so-called radioactive isotopes. The atoms of these particular isotopes undergo spontaneous nuclear breakdown, liberating enormous amounts of energy in the process. This energy appears in the form of high velocity particles (electrons) or as radiant energy (gamma-rays). The very high energy content of these radiations makes it comparatively easy to detect even a single atomic disintegration. The instrument commonly used for this purpose is the Geiger counter which, when it is traversed by an atomic particle or a gamma-ray, releases a sudden minute surge of current; this current is amplified and operates a mechanical counter as each particle arrives.

A pilot experiment carried out in my department will best serve to illustrate the experimental technique used in studying a particular problem. Into an ordinary earthworm was injected a small quantity of radioactive phosphorus (in the form of phosphate in physiological salt solution). The actual amount of radiophosphorus injected is unbelievably small—a total injection of $10^{-12}$ grams of $P^{32}$. Twenty-four hours afterwards, when the material was thoroughly distributed and taking part in the body metabolism, the worm was killed. Various organs or tissues, the nerve cord, the genital tissues, parts of the gut, the body wall, etc., were then isolated by dissection. Each part was completely broken up (homogenized) in distilled water by a high-speed stirrer: and a fraction of the "soup" produced was evaporated to dryness on a small standard nickel tray. The contents of radiophosphorus on each tray were then compared with the help of the Geiger counter. The results are given in Table 1.

TABLE 1

*Uptake of Radiophosphorus by the Earthworm*

Total injection $5\mu C$ of $P^{32}$. Duration of experiment twenty-four hours

| *Specimen* | *Count* |
|---|---|
| Body wall .. .. .. | 1132 per minute |
| Nerve cord .. .. .. | 326 ,, ,, |
| Intestine .. .. .. | 2680 ,, ,, |
| Oesophagus .. .. .. | 7068 ,, ,, |
| Seminal vesicle .. .. | 2260 ,, ,, |

It will be seen that in comparison with the body wall, the nerve cord has a very low uptake of radiophosphorus, while the gut and seminal vesicles show a very high count. The highest count of all is recorded in the oesophagus and later experiments have confirmed this concentration.

Another method of detecting radioactive tracers, which has considerable value in certain cases, is that of auto-radiography. Again, it will be simplest to provide an example from our own work. A dogfish embryo in the egg case was injected in the yolk sac with an approximately similar amount of $P^{32}$ to that used in the case of the earthworm. The embryo was fixed, embedded in paraffin wax and cut into sections $10\mu$ thick. Some of these sections were stained and mounted in the normal way; others were plated out on to a new type of photographic emulsion which is sensitive to the passage of individual electrons, whose tracks show up in the developed emulsion as a chain of black dots. Each track is the product of a single atomic breakdown, and the method is therefore extremely sensitive. Pieces of tissue of only 1/10,000 the size of those used in the previous experiment produce intense blackening of the negative below active parts of the section, and we can detect the radioactivity of organs and structures which are much too small to be dissected out successfully.

The auto-radiograph shows that there is a high concentration of radiophosphorus in the brain, in the pharyngeal region and in the tubules of the pro-nephric kidney. The remainder of the tissues recognizable at this stage (heart, gut and muscles) show a much lower activity.

To understand the meaning of experiments such as these, it is necessary to point out that phosphorus plays a vitally important part in animal and plant metabolism. Its importance in the cell lies in the fact that it occurs in the nucleic acid molecule, and is therefore essential to the production of new cells and new protoplasm, both of which involve initially an increase in the amount of nucleic acid. Though the actual phosphorus content of living cells is not as high, for example, as that of bone, the rate of turnover is so rapid that such cells, particularly if they are growing or reproducing rapidly, exchange in the process very considerable amounts of $P^{32}$ with their environment. The brain shows a high $P^{32}$ uptake in the embryo since there is at this stage in the life-history a very rapid synthesis of new nerve protein as well as a rapid division of nerve cells: the adult nervous system (except when a wounded nerve is regenerated) is comparatively static, as the $P^{32}$ content of the earthworm nerve cord shows.

Phosphorus is also an essential element of the energy-producing mechanisms of the body, whether physical energy, as produced in muscle, or chemical energy, as in secreting cells like those of the oesophagus and kidney. All such sites show a high phosphorus exchange and therefore a high uptake of $P^{32}$.

One of the practical applications of this to medical problems lies in the fact that cancer cells are physiologically in a state of great reproductive activity and growth. They therefore pick up $P^{32}$ more rapidly than the more static surrounding cells. Small areas of surface cancers, undetectable in their early stages by superficial examination, have been detected and located by injecting $P^{32}$ into the patient and then exploring the body surface with a Geiger counter. As one of the promising fields for the treatment of cancer lies in the possibility of a successful early *diagnosis*, this may well prove to be a significant advance.

At first sight, the use of radioactive tracers may appear to offer even more attractive possibilities. One of the most effective methods of treating cancer at present is by X-rays or by radium: the nucleic acid machinery of the cell is extremely sensitive to such radiations. Since $P^{32}$ emits high energy electrons its disintegration is equivalent to a local irradiation of any tissues in which $P^{32}$ is accumulated; the electrons emitted have only a limited penetrating power, so that treatment of a patient with $P^{32}$ ought to be a practical method of selective irradiation of the carcinomatous sites. Unfortunately, this promising scheme breaks down for the same reason as that which limits the value of normal radiotherapy; not only the cancer cells but all other cells which are rapidly reproducing are equally sensitive. In particular, the leucocytes have a high nucleic acid metabolism—they are consequently very sensitive to irradiation and also accumulate $P^{32}$ with great rapidity. X-ray or radium treatment can be limited to areas immediately adjacent to the main site of the disease and some degree of differential dosage of the malignant as opposed to normal tissues is therefore possible. $P^{32}$ accumulates in *all* growing cells, and in practice the leucocytes succumb first.

This feature has, however, been successfully exploited in the treatment of a very rare disease—polycythaemia vera. This disease is characterized by the manufacture of an excessive number of red blood corpuscles; about 60 per cent. of the sufferers show serious haemorrhages or thrombosis, often fatal. The red corpuscles are manufactured from actively growing and reproducing cells in the bone marrow—cells which accumulate $P^{32}$ and destroy themselves in the process. In 124 cases treated by the Mayo Clinic with radioactive phosphorus, satisfactory remissions were obtained in 80 per cent., partial remission in 13 per cent. and only 7 per cent. proved completely intractable. Leukaemia can be similarly dealt with; both lymphatic and myelogenous leukaemia yield with some success to $P^{32}$.

The problem in all these treatments is to localize the site of action of the radioactive material; $P^{32}$ will be taken up most rapidly by the most rapidly growing and reproducing cells. If these happen to be the diseased tissue, there is hope of a successful treatment.

A special case of local accumulation of an element is provided by iodine, which is specifically accumulated by the thyroid in enormous amounts as compared with any other tissue. Radio-iodine has therefore been employed against malignant thyroid growths and even in simple cases of hyperthyroidism. The malignant forms present, however, a secondary problem since it has been found by radioautography that the diseased cells take up less iodine than the healthy cells; the treatment therefore tends to fail because the wrong cells are irradiated. This is a case where radio isotopes have a strong competitor in X-ray therapy.

The use of radioactive tracers in therapeutics is, however, still in its infancy. It has been found possible to prepare radioactive compounds which are deposited by the body at definite sites. Anhydrous chromic phosphate is taken up almost entirely in the liver and spleen. Colloidal radiomanganese dioxide and radioactive gold solutions show possibilities in localized irradiation of lymphoid tissue, but no volume of clinical studies on these has yet been published.

Local irradiation has other attractive possibilities. The rays from different radio elements have widely different penetrating powers. For example, the gamma-rays emitted by radiocobalt have the tremendous penetration of X-rays, and in fact, radiocobalt is now used instead of radium for therapeutic purposes. Beta-rays on the other hand have a limited range, those of $P^{32}$ being stopped by thin glass (very useful for laboratory purposes) and in body tissues have a " half penetration " distance of 1 mm. The effect of their radiation is therefore confined to a few millimeters from the site of origin. Many beta-rays are known of far lower penetrating power. Those of tritium, the radioactive hydrogen isotope, are attracting much attention as material for future studies: their particles will penetrate little more than the diameter of a single cell. If, for example, we can find an organic substance which is specifically accumulated by cancer cells, the " labelling " of this substance with radioactive hydrogen in stable regions of the molecule will permit very high radiation intensity to be concentrated on the diseased cells and on practically no others. The contrast between this and present methods is rather like that between dynamite and D.D.T. as a means of eliminating insect pests!

Less spectacular but equally useful applications of radioactive tracer studies are found in volumetric estimations on normal and pathological circulatory conditions. The dilution of a known amount of radioactive material injected into the blood stream gives a ready measurement of circulating blood volume; by choosing suitable tracers plasma volume, total red cell count, non-cellular fluid phase and even total body water can be rapidly estimated on the living subject. The rate at which an injection becomes manifest at some distant site is a good index of the efficiency of the circulation in the region and very successful observations have been made on the diagnosis of such diseases as arteriosclerosis, and on the efficacy of various remedial treatments.

Tracer technique is in its infancy and is attracting great attention at present. It is, however, merely a new research tool, and as such is liable to misuse or over-emphasis. The attitude of the scientific worker to his problems should be not " What can I do with radioactive tracers? " but " Will tracer elements help me to solve this or that particular problem? " When we have reached that stage, the magic word tracer or radio-element will disappear from the *titles* of our papers and will find its proper place in the small paragraph on methods. In ten years' time, or even less, the present subject will seem a ridiculous topic for a Long Fox lecture—as absurd as discussing the use of the balance in chemical research, or of the scalpel in medical and biological research. The extent to which this attitude has developed will measure our progress in this subject.

